# A novel cognitive neurosurgery approach for supramaximal resection of non-dominant precuneal gliomas: a case report

**DOI:** 10.1007/s00701-023-05755-8

**Published:** 2023-08-19

**Authors:** Garazi Bermúdez, Ileana Quiñones, Alejandro Carrasco, Santiago Gil-Robles, Lucia Amoruso, Emmanel Mandonnet, Manuel Carreiras, Gregorio Catalán, Iñigo Pomposo

**Affiliations:** 1 Neurosurgery Service, Cruces Universitary Hospital, Barakaldo, Spain; 2 Health Research Institute Biocruces Bizkaia, Barakaldo, Spain; 3 University of the Basque Country, UPV/EHU, Bilbao, Spain; 4 Neurobiology of Language, Basque Center On Cognition, Brain and Language, BCBL, Donostia-San Sebastian, Spain; 5 Present Address: IKERBASQUE, Basque Foundation for Science, Bilbao, Spain; 6 Neurosurgery Service, Quironsalud Madrid Universitary Hospital, Madrid, Spain; 7 Lariboisière Hospital, Université Paris 7 Diderot, Paris, France; 8 Frontlab, CNRS UMR 7225, INSERM U1127, Paris, France

**Keywords:** Precuneus, Posterior cingulum, Cognitive awake surgery

## Abstract

Despite mounting evidence pointing to the contrary, classical neurosurgery presumes many cerebral regions are non-eloquent, and therefore, their excision is possible and safe. This is the case of the precuneus and posterior cingulate, two interacting hubs engaged during various cognitive functions, including reflective self-awareness; visuospatial and sensorimotor processing; and processing social cues. This inseparable duo ensures the cortico-subcortical connectivity that underlies these processes. An adult presenting a right precuneal low-grade glioma invading the posterior cingulum underwent awake craniotomy with direct electrical stimulation (DES). A supramaximal resection was achieved after locating the superior longitudinal fasciculus II. During surgery, we found sites of positive stimulation for line bisection and mentalizing tests that enabled the identification of surgical corridors and boundaries for lesion resection. When post-processing the intraoperative recordings, we further identified areas that positively responded to DES during the trail-making and mentalizing tests. In addition, a clear worsening of the patient’s self-assessment ability was observed throughout the surgery. An awake cognitive neurosurgery approach allowed supramaximal resection by reaching the cortico-subcortical functional limits. The mapping of complex functions such as social cognition and self-awareness is key to preserving patients’ postoperative cognitive health by maximizing the ability to resect the lesion and surrounding areas.

**Supplementary Information:**

The online version contains supplementary material available at 10.1007/s00701-023-05755-8.

## Introduction

The precuneus, located in the mesial aspect of the superior parietal lobule, is often considered by neurosurgeons as a non-eloquent area [14, 26]. Nevertheless, patients with tumors affecting this parieto-mesial area have been reported to experience mild cognitive disturbances after surgery [11, 31]. Its anatomical landmarks are anteriorly the postcentral sulcus, inferiorly the dorsal posterior cingulum, and posteriorly the parieto-occipital sulcus. Although its functionality remains unclear, a complex associative role is presumed due to multiple connections with different cortical areas [1, 27]. Its connectivity profile explains its involvement in various cognitive functions including reflective self-awareness; visuospatial and sensorimotor processing; episodic memory; and processing social cues [1, 6, 27, 31]. Notably, the functional connectivity between posterior cingulate/precuneus and hypothalamus, thalamus, ventromedial prefrontal cortex, superior temporal gyrus, and cerebellum places this duo at the core of both the default-mode and frontoparietal attentional networks [1, 15]. Additionally, its connection to the posterior cingulum, which is implicated in several aspects of social cognition, further underscores its importance [6, 15].

Recent research has shed light on the posterior cingulate cortex, highlighting its unique anatomical and physiological properties, as well as its significant contributions to supramodal cognitive functions and brain disorders [7, 22]. In light of these findings, Foster et al. [6] propose a tripartite perspective on this region. According to their review, the dorsal part is implicated in executive control functions, the ventral portion supports memory processes, and the retrosplenial cortex plays a crucial role in visuospatial abilities. However, the use of neuropsychological tests assessing these complex cognitive functions in intraoperative functional mapping as guidance for the safe resection of precuneal/posterior cingulate lesions has been limited.

This study aimed to develop an intraoperative protocol for mapping the precuneus/posterior cingulum area, considering its involvement in the aforementioned cognitive functions, and evaluate its effectiveness in a clinical case of a grade II right oligodendroglioma that affected both the precuneus and dorsal posterior cingulum. Furthermore, the comprehensive investigations conducted in this case underscored the functional and connectivity aspects that should be considered when operating on lesions affecting these regions.

## Materials and methods

### Clinical case

**Fig. 1:**
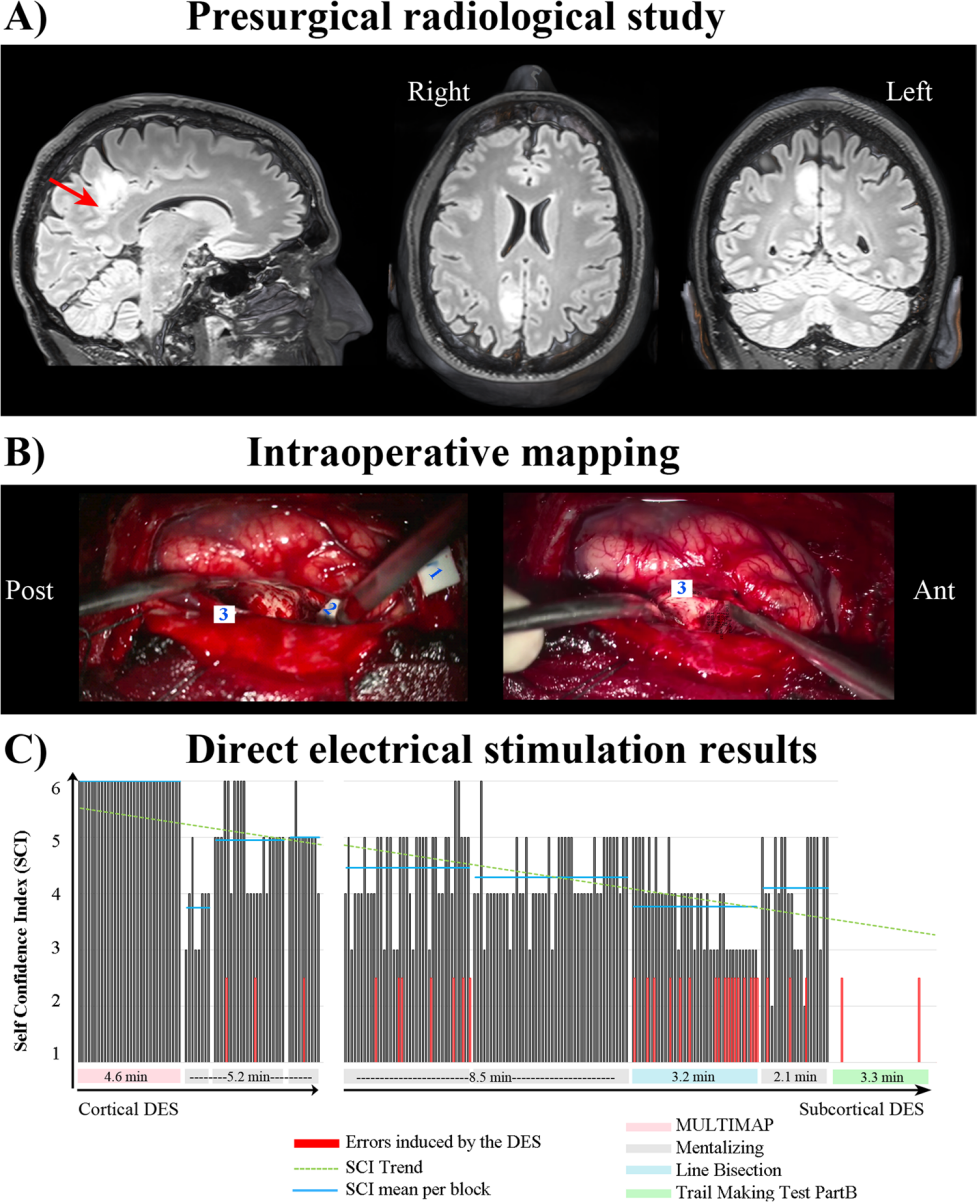
Radiological study and intraoperative mapping. **A** Presurgical 3 T MRI study showing a right precuneal tumor with mild posterior dorsal cingulum invasion (radiological view). **B** Surgical view of the resection cavity with DES response sites labeled as 1 for the right sensory cortex (lower limb), 2 for the thalamocortical tract, and 3 for the SLF II. **C** Bar graph showing the sequence of tasks performed during the intraoperative stimulation phase. The x-axis represents the trials per task, while the y-axis indicates the self-confidence index reported by the patients (from 1 to 6). To assist in interpretation, gray vertical lines denote the trials conducted during the DES phase. Additionally, the graph employs colored horizontal bars at the bottom to represent different tasks, with the duration of each task indicated by numbers within the bars, measured in minutes. The consecutive appearance of bars in the same color signifies multiple repetitions of the corresponding task. Notably, the occurrence of behavioral errors associated with DES is represented by a red line. In particular, deviations exceeding 5% to the left in both the line bisection task and TMT-Part B are considered errors. To further aid understanding, the graph incorporates a light blue dotted line indicating the overall decrease in the self-confidence index. Furthermore, the green lines depict the mean self-confidence index for each stimulation block, offering insights into the patient’s metacognitive state during the stimulation phase

A 39-year-old right-handed man was diagnosed with an incidental 4.6 cc right anterior precuneal lesion after consulting for long-term hypoacusia with a left predominance (Fig. 1A). A biopsy showed a grade II oligodendroglioma.^1^

### Presurgical radiological study

The patient underwent an MRI session in a 3 T Siemens Magnetom Prisma Fit scanner (Siemens AG, Erlangen, Germany). High-resolution T1- and T2-weighted images were acquired with a 3D ultrafast gradient echo MPRAGE pulse sequence using a 64-channel head coil covering 160 contiguous axial slices with a voxel resolution of 1 × 1 × 1 mm^3^. This protocol showed a T1-weighted hypointense and T2-weighted hyperintense cortical lesion, without enhancement after gadolinium injection. Diffusion-weighted sequences were acquired along 105 independent directions with a *b* value of 900 s/mm^2^.

### Surgery

Resective awake surgery, conducted under conscious sedation with dexmedetomidine, was provided for online monitoring of cognitive functions in real-time. The primary objective was to accurately identify functionally significant structures (i.e., positive response sites) and optimize the extent of volume resection while minimizing the risk of postoperative functional impairments. A right parasagittal parietal craniotomy was performed, exposing the superior parietal lobule and upper sensorimotor cortices. Cortical and subcortical mapping were conducted by direct electrical stimulation (DES) at 60 Hz delivered with human use certified intraoperative equipment (NimEclipse®, Medtronic), using a bipolar stimulation probe (Inomed, fork probe 45-mm straight, ball tip diameter 2 mm, tip to tip distance 8 mm). After cortical stimulation at 2.5 mA, the primary motor cortex controlling the arm was found. Usually, an interhemispheric parafalcine approach would be considered to reach the lesion, either from the contralateral or ipsilateral side. However, in this case, a transcortical parasagittal approach was elected, since the mapping of the superior parietal lobule surrounding the lesion was negative. This approach minimized the risk of damaging cortical veins entering the superior longitudinal sinus and allows better exposure of the lesion, facilitating gross total resection. The procedure lasted for 4 h and 45 min, with the patient being awake for a total of ~ 90 min. Throughout this period, there were alternating periods of stimulation and rest. Despite being advised to rest, the patient engaged in open conversation during the rest periods. Cortical stimulation was initially employed to determine the entry point, while subcortical stimulation was utilized upon reaching specific functional limits (see Fig. 1B for an intracortical timeline).

### Cortical and subcortical intraoperative functional mapping

Functional mapping was performed while the patient engaged in picture-naming, mentalizing, trail-making, and line bisection tests (for details see Table 1 and Video 1). During the pre-stimulation phase, the patient accurately completed all the tasks. At the cortical level, we started with the classic picture-naming test [8], after which we used the mentalizing task [3] for tumor resection. At the subcortical level, we conducted line bisection [5, 28], trail-making [13, 18], and mentalizing tests (see Fig. 1B for a timeline of the intracortical stimulation). In addition, throughout the different surgery stages, we employed the self-confidence index to assess the patient’s self-awareness. In each trial, the patient had to evaluate his performance using a Likert-scale ranging from 1 to 6, where 1 was “I am very doubtful of the answer I gave” and 6 indicated “I am absolutely certain of the answer I gave”.

**Table 1:**
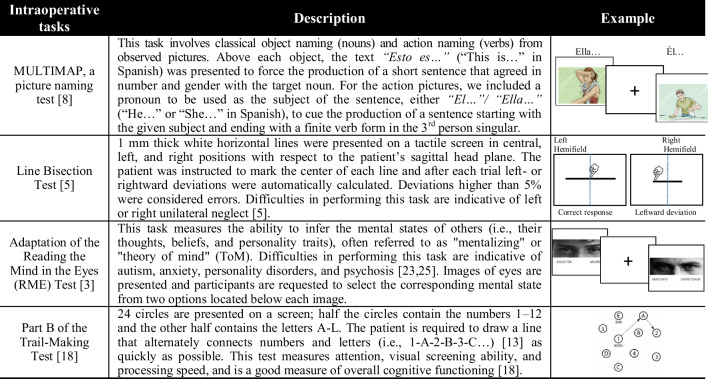
Description of the neuropsychological tests

## Results

### Intraoperative functional mapping

**Fig. 2:**
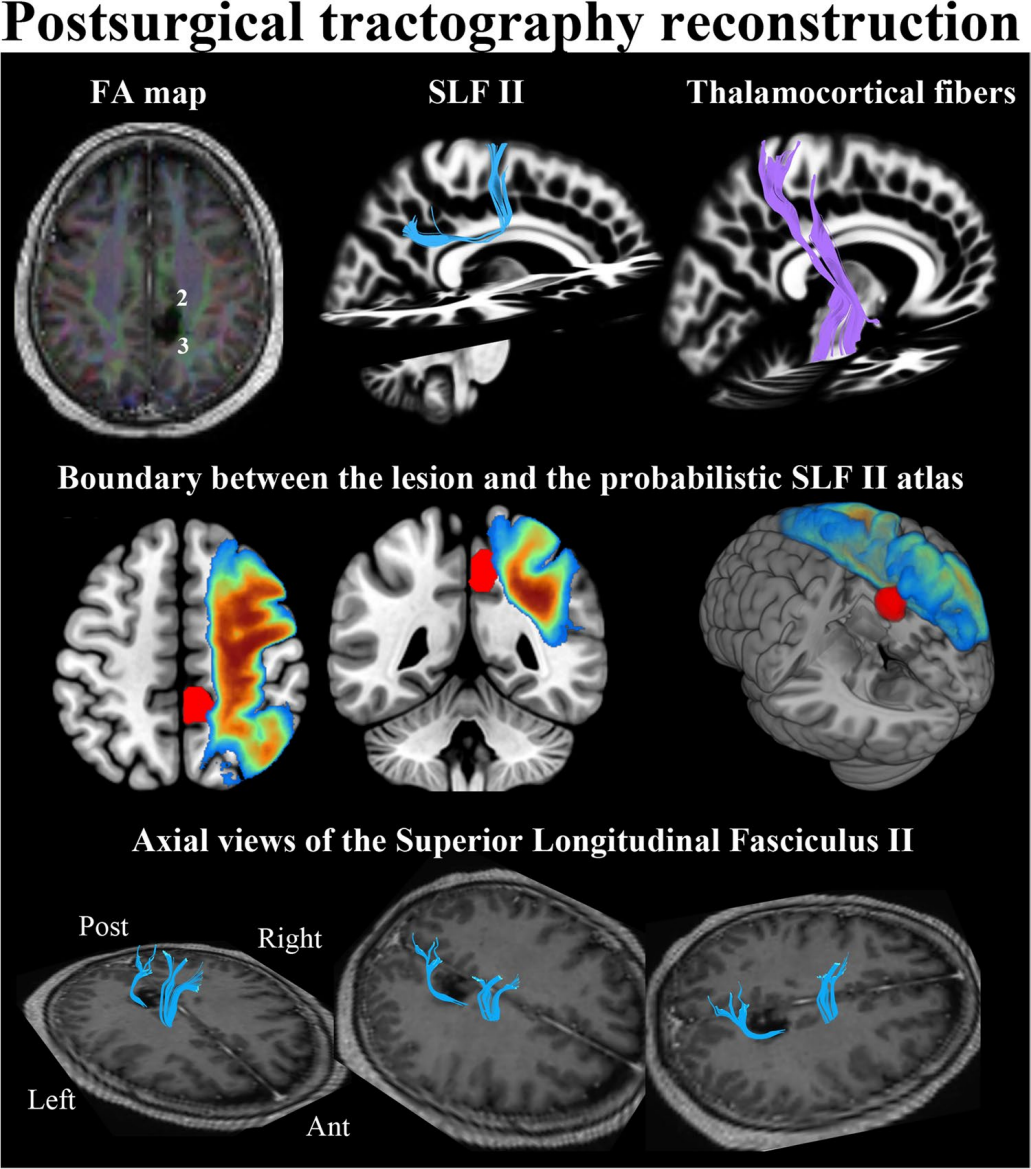
Postoperative 3 T MRI study. The upper part of the figure displays various imaging representations related to the surgical procedure. On the left, a postoperative fractional anisotropy (FA) color map illustrates the surgical cavity, with positively stimulated points labeled as 2 and 3, as identified during intraoperative assessment. In the upper right section, a tractographic study reveals the presence of the SLF II depicted in blue, along with the sensory portion of the right thalamocortical tract shown in violet. These fibers surround the tumor, with SLF II situated laterally and the thalamocortical tract anteriorly. The central panel presents a probabilistic atlas of SLF II [24] and the 3D tumor reconstruction superimposed on the MNI template. It is worth noting that the tumor extends towards the lateral aspect of SLF II. DSIstudio, a software platform for visualization and analysis of multi-modality brain data, was used to visualize the anisotropy data, track fibers, and coregister T1-weighted MRI with DTI

During the pre-stimulation phase, the patient accurately completed all the tasks. At the cortical level, as expected, no positive stimulation sites were found for the picture-naming and mentalizing tests. However, at the subcortical level, in the inferolateral boundary of the resection cavity, the mentalizing, line bisection, and TMT-Part B tests were transiently disturbed in the area marked as 3 in Fig. 1B and Fig. 2. Conducting diffusion tensor magnetic resonance tractography on preoperative MRI — coregistered with the postoperative study — enabled us to precisely identify these fibers as part of the superior longitudinal fasciculus (SLF) II, a major component of the SLF, that connects the caudal inferior parietal region to the dorsolateral frontal cortex (Fig. 2). Moreover, the self-assessment task showed an overall decrease in self-confidence throughout the surgery: at the beginning of the surgery, the self-confidence index was always 6 (the maximum) whereas, at the end of the resection, it varied between 3 and 5 (see Fig. 1B).

### Postoperative neuropsychological assessment

Immediately after surgery, the patient only reported transient dysphasia for proper names, with total recovery by discharge at postoperative day four. The 1-month postoperative neuropsychological assessment showed no cognitive impairments.

### Postsurgical radiological study

The patient underwent an MRI session following the protocol designed for the presurgical study. As observed in Fig. 1, a supramaximal resection was achieved (cavity volume = 7.4 cc, EOR ~ 160%) reaching both anatomical and functional limits located by subcortical stimulation: (a) anteriorly, postcentral sulcus, and thalamocortical tract (label 2 in Fig. 2); (b) laterally, SLF II (label 3 in Fig. 2); (c) medially, falx cerebri; (d) inferiorly, corpus callosum; (e) posteriorly, the lower end of the medial part of the parietooccipital sulcus. Figure 2 shows how the subcortical white matter pathways that ensure connectivity between the parietal and frontal lobes were preserved.

## Discussion

As links between neurosurgery and neurosciences are consolidated, new concepts, such as brain connectomics, are being incorporated into neurosurgical practice. The safe excision of so-called “*non-eloquent”* regions such as the precuneus and the posterior cingulum provides a paradigmatic example of these recent advances. As many studies based on neuroimaging techniques have demonstrated the posterior cingulate/precuneus represents a functionally important associative area [6, 7, 22, 27, 31], an awake cognitive neurosurgery approach was considered critical for the removal of a tumor located in this area. Thanks to appropriate neuropsychological tasks used during the intraoperative mapping, it has been possible to identify the functional boundaries of the right posterior cingulum/precuneus, hence allowing a supramaximal tumor resection without postoperative cognitive impairments.

Several fMRI studies demonstrated the involvement of the precuneus and posterior cingulum, generally co-activated, in various functions ranging from reflective self-awareness, visuospatial, and sensorimotor processing, to processing social cues [11, 15, 23]. Disruptions in the posterior cingulum in both hemispheres have been reported to alter both self-consciousness and consciousness of the external environment, including personality disorders such as derealization or depersonalization [2, 11, 12]. Recently, the role of the anterodorsal part of the precuneus in sensorimotor processing has been demonstrated by Herbet et al. [12], as different body awareness disorders were elicited after lesions in that zone [17, 23]. This parietal area, which lies at the core of the default mode and frontoparietal attentional networks [1], has been reported as a “connector hub” ensuring the cortico-subcortical connectivity that underlies the information flow between posterior regions and hypothalamus, thalamus, ventromedial prefrontal cortex, superior temporal gyrus, and cerebellum [4, 32].

This connectivity is, in part, guaranteed by the SLF II, whose stem is located lateral to corona radiata. This tract can be found deep in the inferior parietal lobule, below U fibers in the intraparietal sulcus. It is part of the dorsal attention pathway and plays an important role in visuospatial awareness [5, 16]. Damage to this tract has previously been also associated with social and emotional impairments [20, 21]. Tumoral infiltration of the right SLF II has been correlated with postoperative declines in mentalizing performance in glioma patients [10]. The precise structure of SLF tracts I, II, and III remains unclear, and their segmentation is not fully understood, primarily relying on differences in observed intraoperative neurological deficits during awake craniotomies [29]. In our current case, we identified a slowing of the motor response in the anterior part of the cavity, which we interpreted as the boundary between the lesion and the thalamocortical pathway (indicated in purple in Fig. 2). Conversely, functional delimitation of SLF II was achieved by stimulating the posterior lateral wall of the cavity (corresponding to point 3 in Figs. 1 and 2). The depth of the intraparietal sulcus served as the anatomical reference for this point. Notably, at this location, we observed a convergence of visuospatial abilities (measured with the line bisection and trial-making tests) and mentalizing or theory of mind processes (measured with the mentalizing task). Stimulation of the posterolateral (closer to the midline) wall of the cavity did not result in motor slowing, which would have indicated the functional boundary of SLF I. Additionally, we did not encounter any specific language deficits that would indicate the functional limit of SLF III. Previous studies have extensively reported transient errors in the line bisection task associated with the SLF II stimulation [19, 28, 30]. This contralateral bias caused by the temporal disconnection of SLF II reflects the involvement of this tract in tasks requiring visuospatial integration, which is crucial for accurate performance on the trial-making test. Hence, we found that these two functions coexist at the same point.

Based on this multifunctionality, we designed a safe surgical protocol for intraoperative mapping consisting of four neuropsychological tests. The picture-naming test was employed to monitor any fluctuations in attentional levels occurring during surgery. Line bisection, trail-making, and mentalizing tests were used to respectively measure visuospatial awareness [5], attention and cognitive flexibility [18], and the ability to infer the mental states of others [3, 25]. Furthermore, the patient’s self-awareness was monitored by utilizing the self-confidence index^6^. All these tests have been previously used to investigate the brain mechanisms underlying cognition during awake craniotomies [9, 17, 23]; however, to the best of our knowledge, this is the first time their use has been successfully coordinated for the intraoperative functional mapping to guide safe surgical resection of a precuneal lesion. We took advantage of the DES capacity to map, in real-time, the functioning of cortico-subcortical networks: electrical stimulation of subcortical bundles induced clear disruptions in the mentalizing, trail-making, and line bisection tests. Although the tumor was confined to the precuneus and the dorsal part of the cingulum without reaching the SLF II laterally, awakening the patient was crucial to extend the resection both anteriorly and laterally, until the sensory pathway and SLF II were identified. This way, a supramaximal resection was safely achieved, with a positive impact on the patient’s survival, global health, and quality of life as he presented no cognitive impairments 1 month after the surgery.

## Conclusions

Due to the increased life expectancy of low-grade glioma patients, it is desirable to improve neurosurgical approaches to further increase their quality of life. Although standardized protocols are followed for the surgical treatment of brain tumors located in functional areas of the language, motor, and somatosensory networks, other cognitive functions, such as visual awareness, attention, cognitive flexibility, or our ability to comprehend, and express emotional states have been overlooked. The present study suggests that this bias can be mitigated through the creation of intervention protocols aimed at mapping hitherto neglected cognitive domains such as those explored here. Increasing our knowledge of connectomics and higher-level cognitive functioning may open new avenues and insights to overcome misconceptions among neurosurgeons about non-eloquent areas. An awake cognitive neurosurgery approach constitutes an excellent and safe tool for preserving cognitive functions while performing/achieving supramaximal resection of a tumor, as otherwise non-identifiable functional limits can be reached.

### Supplementary Information

Below is the link to the electronic supplementary material.

Supplementary file1 Supplementary Video 1.*Audiovisual presentation of the clinical case.* The video summarized an awake cognitive neurosurgery approach that allowed supramaximal resection of a non-dominant precuneal low-grade glioma. Functional mapping was performed while the patient engaged in picture naming, mentalizing, trail-making, and line bisection tests. Sites of positive stimulation for line bisection and mentalizing tests were identified enabling the identification of surgical corridors and boundaries for lesion resection. A clear worsening of the patient’s self-assessment ability was observed throughout the surgery. (MOV 309806 KB)

## Data Availability

The data presented in this study as well as the cognitive tasks and experimental stimuli used during the surgery are available on request from the corresponding author. The data are not publicly available due to the data-sharing policies of the different institutions involved.

## Footnotes

1 After undergoing the biopsy procedure, the patient experienced sensitivity issues in their right hand that improved with rehabilitation.
